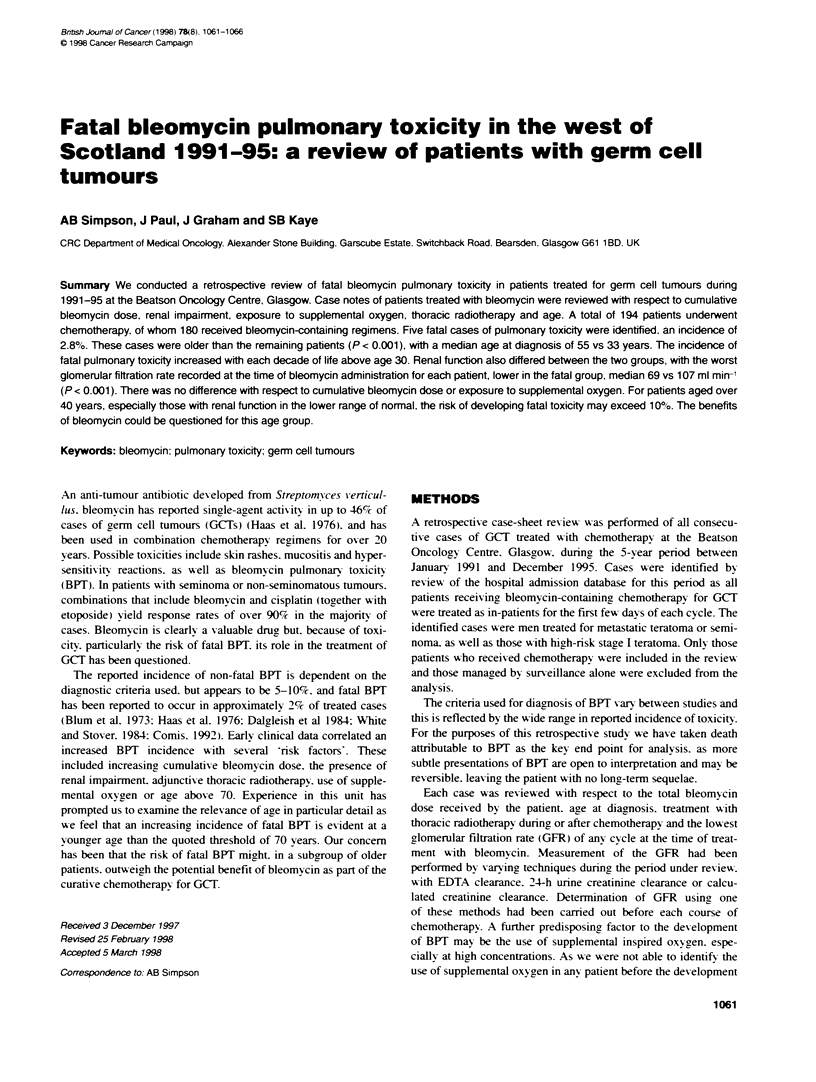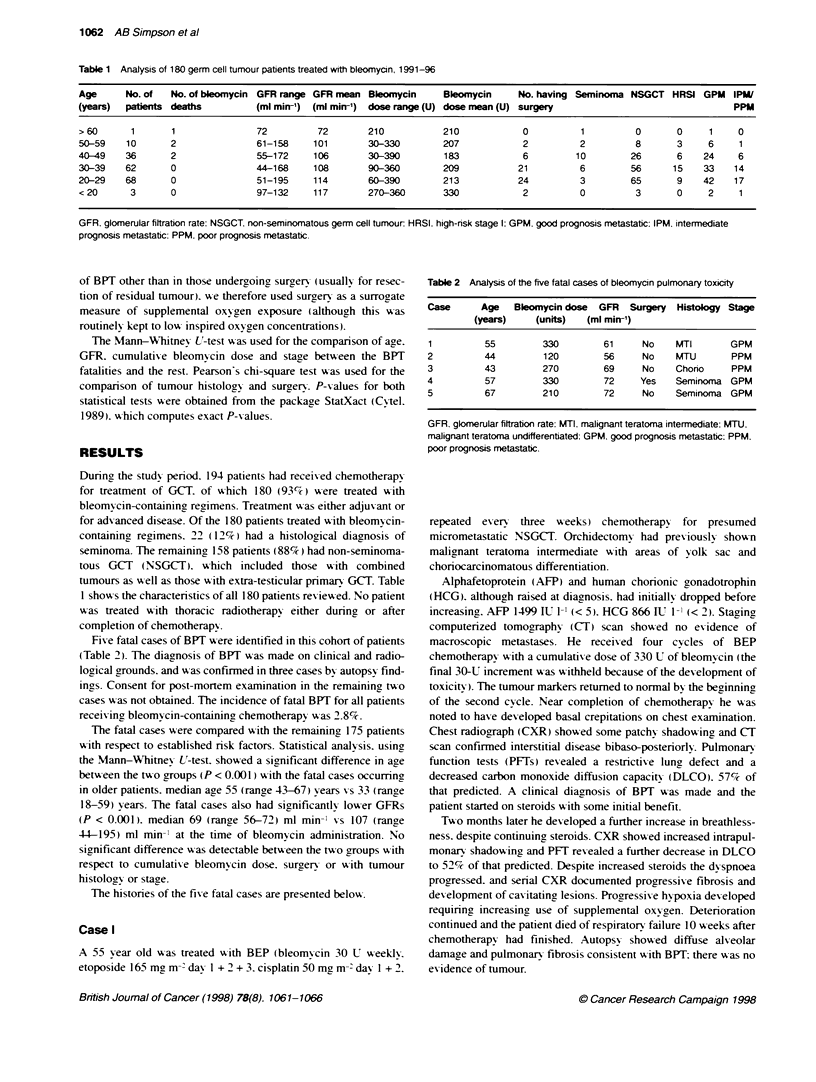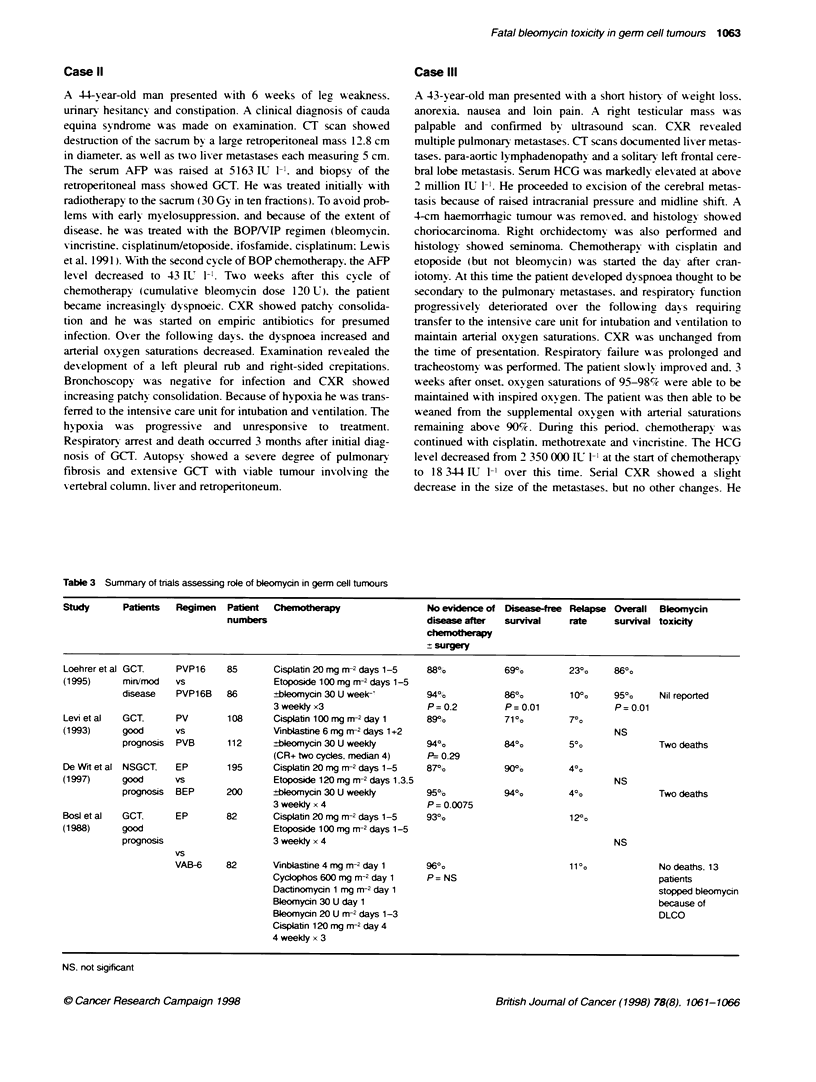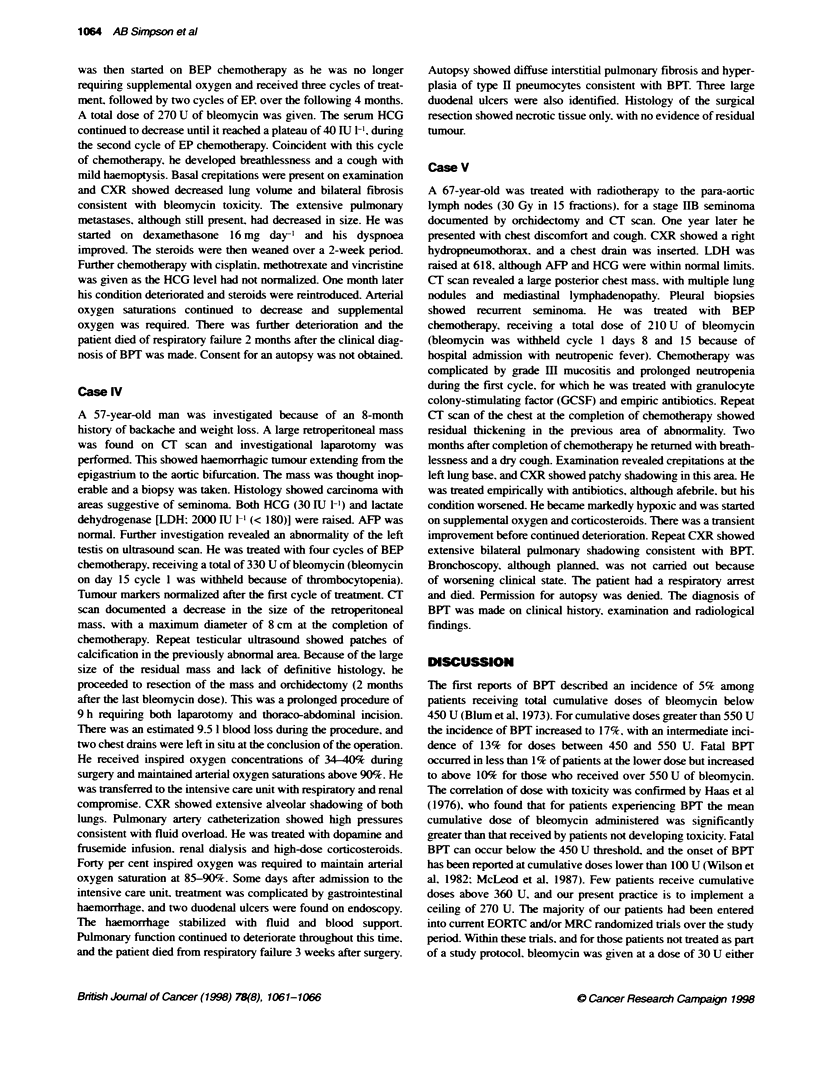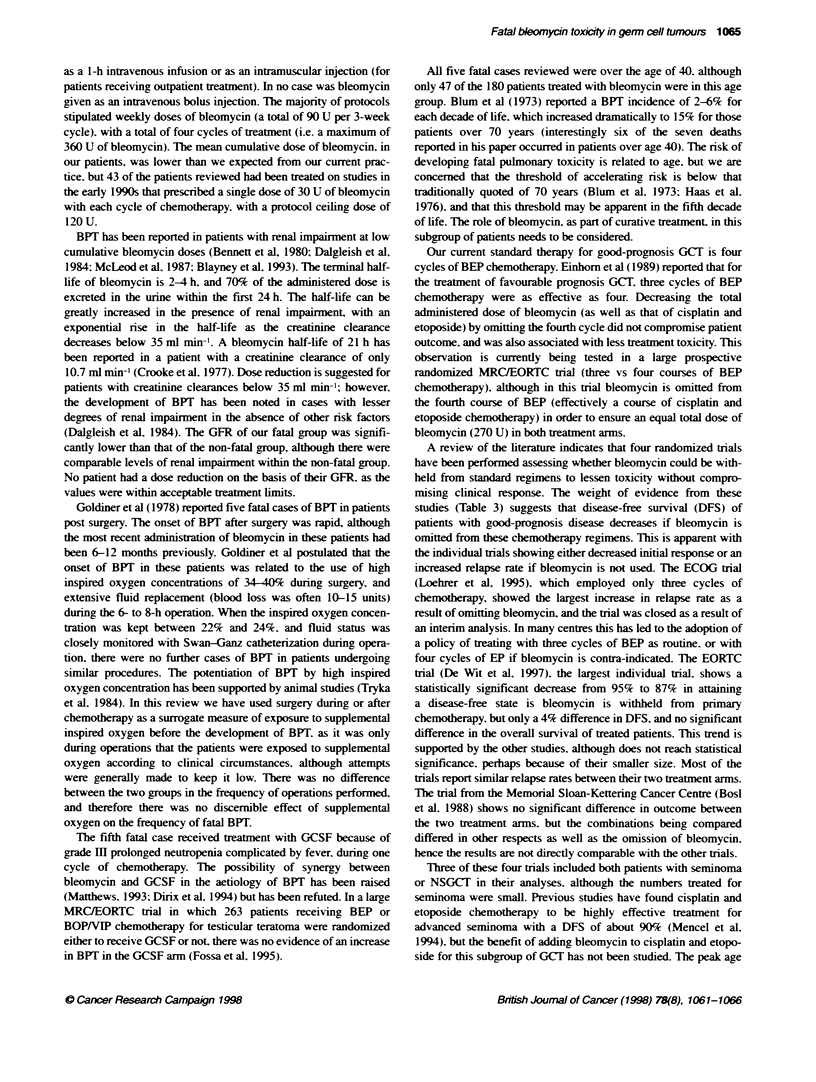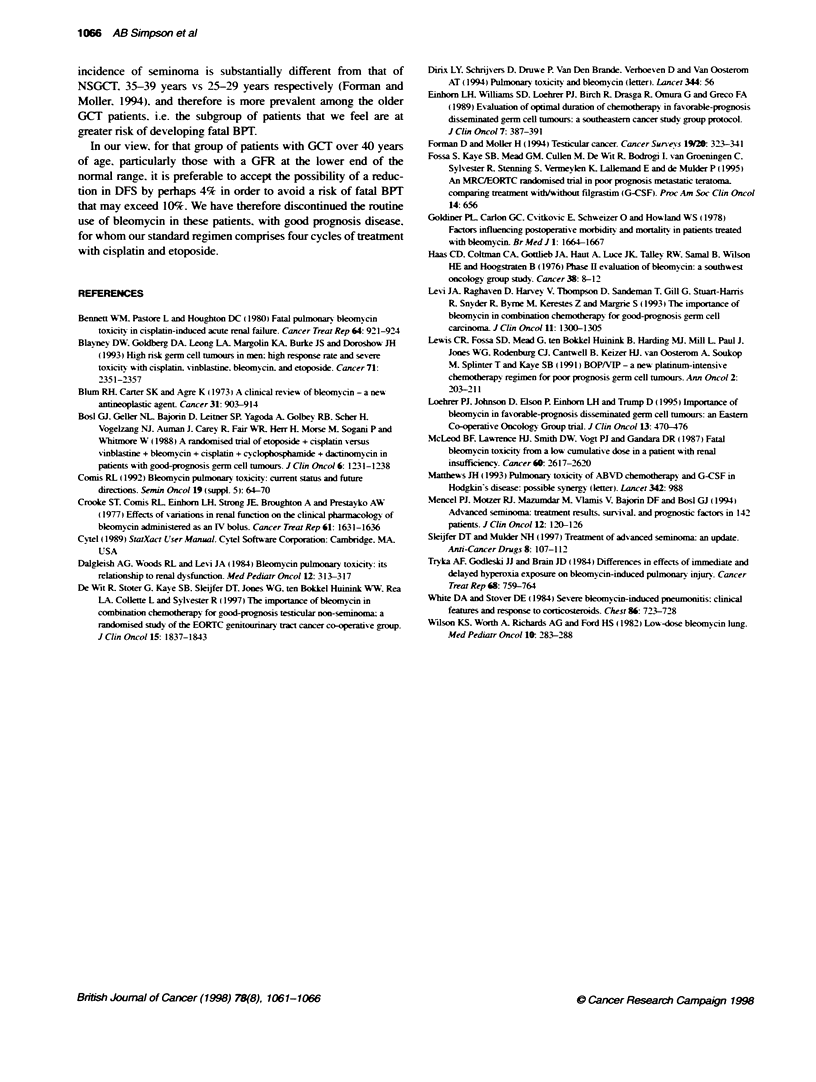# Fatal bleomycin pulmonary toxicity in the west of Scotland 1991-95: a review of patients with germ cell tumours.

**DOI:** 10.1038/bjc.1998.628

**Published:** 1998-10

**Authors:** A. B. Simpson, J. Paul, J. Graham, S. B. Kaye

**Affiliations:** CRC Department of Medical Oncology, Glasgow, UK.

## Abstract

We conducted a retrospective review of fatal bleomycin pulmonary toxicity in patients treated for germ cell tumours during 1991-95 at the Beatson Oncology Centre, Glasgow. Case notes of patients treated with bleomycin were reviewed with respect to cumulative bleomycin dose, renal impairment, exposure to supplemental oxygen, thoracic radiotherapy and age. A total of 194 patients underwent chemotherapy, of whom 180 received bleomycin-containing regimens. Five fatal cases of pulmonary toxicity were identified, an incidence of 2.8%. These cases were older than the remaining patients (P < 0.001), with a median age at diagnosis of 55 vs 33 years. The incidence of fatal pulmonary toxicity increased with each decade of life above age 30. Renal function also differed between the two groups, with the worst glomerular filtration rate recorded at the time of bleomycin administration for each patient, lower in the fatal group, median 69 vs 107 ml min(-1) (P < 0.001). There was no difference with respect to cumulative bleomycin dose or exposure to supplemental oxygen. For patients aged over 40 years, especially those with renal function in the lower range of normal, the risk of developing fatal toxicity may exceed 10%. The benefits of bleomycin could be questioned for this age group.


British Journal of Cancer (1998) 78(8), 1061–1066
© 1998 Cancer Research Campaign

### Fatal bleomycin pulmonary toxicity in the west of Scotland 1991–95: a review of patients with germ cell tumours

**AB Simpson, J Paul, J Graham and SB Kaye**

CRC Department of Medical Oncology, Alexander Stone Building, Garscube Estate, Switchback Road, Bearsden, Glasgow G61 1BD, UK

**Summary** We conducted a retrospective review of fatal bleomycin pulmonary toxicity in patients treated for germ cell tumours during 1991–95 at the Beatson Oncology Centre, Glasgow. Case notes of patients treated with bleomycin were reviewed with respect to cumulative bleomycin dose, renal impairment, exposure to supplemental oxygen, thoracic radiotherapy and age. A total of 194 patients underwent chemotherapy, of whom 180 received bleomycin-containing regimens. Five fatal cases of pulmonary toxicity were identified, an incidence of 2.8%. These cases were older than the remaining patients ($P < 0.001$), with a median age at diagnosis of 55 vs 33 years. The incidence of fatal pulmonary toxicity increased with each decade of life above age 30. Renal function also differed between the two groups, with the worst glomerular filtration rate recorded at the time of bleomycin administration for each patient, lower in the fatal group, median 69 vs 107 ml $min^{-1}$ ($P < 0.001$). There was no difference with respect to cumulative bleomycin dose or exposure to supplemental oxygen. For patients aged over 40 years, especially those with renal function in the lower range of normal, the risk of developing fatal toxicity may exceed 10%. The benefits of bleomycin could be questioned for this age group.

**Keywords:** bleomycin; pulmonary toxicity; germ cell tumours

An anti-tumour antibiotic developed from *Streptomyces verticullus*, bleomycin has reported single-agent activity in up to 46% of cases of germ cell tumours (GCTs) (Haas et al, 1976), and has been used in combination chemotherapy regimens for over 20 years. Possible toxicities include skin rashes, mucositis and hypersensitivity reactions, as well as bleomycin pulmonary toxicity (BPT). In patients with seminoma or non-seminomatous tumours, combinations that include bleomycin and cisplatin (together with etoposide) yield response rates of over 90% in the majority of cases. Bleomycin is clearly a valuable drug but, because of toxicity, particularly the risk of fatal BPT, its role in the treatment of GCT has been questioned.

The reported incidence of non-fatal BPT is dependent on the diagnostic criteria used, but appears to be 5–10%, and fatal BPT has been reported to occur in approximately 2% of treated cases (Blum et al, 1973; Haas et al, 1976; Dalgleish et al 1984; White and Stover, 1984; Comis, 1992). Early clinical data correlated an increased BPT incidence with several 'risk factors'. These included increasing cumulative bleomycin dose, the presence of renal impairment, adjunctive thoracic radiotherapy, use of supplemental oxygen or age above 70. Experience in this unit has prompted us to examine the relevance of age in particular detail as we feel that an increasing incidence of fatal BPT is evident at a younger age than the quoted threshold of 70 years. Our concern has been that the risk of fatal BPT might, in a subgroup of older patients, outweigh the potential benefit of bleomycin as part of the curative chemotherapy for GCT.

*Received 3 December 1997*
*Revised 25 February 1998*
*Accepted 5 March 1998*

*Correspondence to:* AB Simpson

### METHODS

A retrospective case-sheet review was performed of all consecutive cases of GCT treated with chemotherapy at the Beatson Oncology Centre, Glasgow, during the 5-year period between January 1991 and December 1995. Cases were identified by review of the hospital admission database for this period as all patients receiving bleomycin-containing chemotherapy for GCT were treated as in-patients for the first few days of each cycle. The identified cases were men treated for metastatic teratoma or seminoma, as well as those with high-risk stage I teratoma. Only those patients who received chemotherapy were included in the review and those managed by surveillance alone were excluded from the analysis.

The criteria used for diagnosis of BPT vary between studies and this is reflected by the wide range in reported incidence of toxicity. For the purposes of this retrospective study we have taken death attributable to BPT as the key end point for analysis, as more subtle presentations of BPT are open to interpretation and may be reversible, leaving the patient with no long-term sequelae.

Each case was reviewed with respect to the total bleomycin dose received by the patient, age at diagnosis, treatment with thoracic radiotherapy during or after chemotherapy and the lowest glomerular filtration rate (GFR) of any cycle at the time of treatment with bleomycin. Measurement of the GFR had been performed by varying techniques during the period under review, with EDTA clearance, 24-h urine creatinine clearance or calculated creatinine clearance. Determination of GFR using one of these methods had been carried out before each course of chemotherapy. A further predisposing factor to the development of BPT may be the use of supplemental inspired oxygen, especially at high concentrations. As we were not able to identify the use of supplemental oxygen in any patient before the development

**Table 1** Analysis of 180 germ cell tumour patients treated with bleomycin, 1991–96

| Age (years) | No. of patients | No. of bleomycin deaths | GFR range (ml min$^{-1}$) | GFR mean (ml min$^{-1}$) | Bleomycin dose range (U) | Bleomycin dose mean (U) | No. having surgery | Seminoma | NSGCT | HRSI | GPM | IPM/PPM |
|---|---|---|---|---|---|---|---|---|---|---|---|---|
| > 60 | 1 | 1 | 72 | 72 | 210 | 210 | 0 | 1 | 0 | 0 | 1 | 0 |
| 50–59 | 10 | 2 | 61–158 | 101 | 30–330 | 207 | 2 | 2 | 8 | 3 | 6 | 1 |
| 40–49 | 36 | 2 | 55–172 | 106 | 30–390 | 183 | 6 | 10 | 26 | 6 | 24 | 6 |
| 30–39 | 62 | 0 | 44–168 | 108 | 90–360 | 209 | 21 | 6 | 56 | 15 | 33 | 14 |
| 20–29 | 68 | 0 | 51–195 | 114 | 60–390 | 213 | 24 | 3 | 65 | 9 | 42 | 17 |
| < 20 | 3 | 0 | 97–132 | 117 | 270–360 | 330 | 2 | 0 | 3 | 0 | 2 | 1 |

GFR, glomerular filtration rate; NSGCT, non-seminomatous germ cell tumour; HRSI, high-risk stage I; GPM, good prognosis metastatic; IPM, intermediate prognosis metastatic; PPM, poor prognosis metastatic.

of BPT other than in those undergoing surgery (usually for resection of residual tumour), we therefore used surgery as a surrogate measure of supplemental oxygen exposure (although this was routinely kept to low inspired oxygen concentrations).

The Mann–Whitney *U*-test was used for the comparison of age, GFR, cumulative bleomycin dose and stage between the BPT fatalities and the rest. Pearson's chi-square test was used for the comparison of tumour histology and surgery. *P*-values for both statistical tests were obtained from the package StatXact (Cytel, 1989), which computes exact *P*-values.

### RESULTS

During the study period, 194 patients had received chemotherapy for treatment of GCT, of which 180 (93%) were treated with bleomycin-containing regimens. Treatment was either adjuvant or for advanced disease. Of the 180 patients treated with bleomycin-containing regimens, 22 (12%) had a histological diagnosis of seminoma. The remaining 158 patients (88%) had non-seminomatous GCT (NSGCT), which included those with combined tumours as well as those with extra-testicular primary GCT. Table 1 shows the characteristics of all 180 patients reviewed. No patient was treated with thoracic radiotherapy either during or after completion of chemotherapy.

Five fatal cases of BPT were identified in this cohort of patients (Table 2). The diagnosis of BPT was made on clinical and radiological grounds, and was confirmed in three cases by autopsy findings. Consent for post-mortem examination in the remaining two cases was not obtained. The incidence of fatal BPT for all patients receiving bleomycin-containing chemotherapy was 2.8%.

The fatal cases were compared with the remaining 175 patients with respect to established risk factors. Statistical analysis, using the Mann–Whitney *U*-test, showed a significant difference in age between the two groups ($P < 0.001$) with the fatal cases occurring in older patients, median age 55 (range 43–67) years vs 33 (range 18–59) years. The fatal cases also had significantly lower GFRs ($P < 0.001$), median 69 (range 56–72) ml min$^{-1}$ vs 107 (range 44–195) ml min$^{-1}$ at the time of bleomycin administration. No significant difference was detectable between the two groups with respect to cumulative bleomycin dose, surgery or with tumour histology or stage.

The histories of the five fatal cases are presented below.

**Table 2** Analysis of the five fatal cases of bleomycin pulmonary toxicity

| Case | Age (years) | Bleomycin dose (units) | GFR (ml min$^{-1}$) | Surgery | Histology | Stage |
|---|---|---|---|---|---|---|
| 1 | 55 | 330 | 61 | No | MTI | GPM |
| 2 | 44 | 120 | 56 | No | MTU | PPM |
| 3 | 43 | 270 | 69 | No | Chorio | PPM |
| 4 | 57 | 330 | 72 | Yes | Seminoma | GPM |
| 5 | 67 | 210 | 72 | No | Seminoma | GPM |

GFR, glomerular filtration rate; MTI, malignant teratoma intermediate; MTU, malignant teratoma undifferentiated; GPM, good prognosis metastatic; PPM, poor prognosis metastatic.

### Case I

A 55 year old was treated with BEP (bleomycin 30 U weekly, etoposide 165 mg m$^{-2}$ day 1 + 2 + 3, cisplatin 50 mg m$^{-2}$ day 1 + 2, repeated every three weeks) chemotherapy for presumed micrometastatic NSGCT. Orchidectomy had previously shown malignant teratoma intermediate with areas of yolk sac and choriocarcinomatous differentiation.

Alphafetoprotein (AFP) and human chorionic gonadotrophin (HCG), although raised at diagnosis, had initially dropped before increasing, AFP 1499 IU l$^{-1}$ (< 5), HCG 866 IU l$^{-1}$ (< 2). Staging computerized tomography (CT) scan showed no evidence of macroscopic metastases. He received four cycles of BEP chemotherapy with a cumulative dose of 330 U of bleomycin (the final 30-U increment was withheld because of the development of toxicity). The tumour markers returned to normal by the beginning of the second cycle. Near completion of chemotherapy he was noted to have developed basal crepitations on chest examination. Chest radiograph (CXR) showed some patchy shadowing and CT scan confirmed interstitial disease bibaso-posteriorly. Pulmonary function tests (PFTs) revealed a restrictive lung defect and a decreased carbon monoxide diffusion capacity (DLCO), 57% of that predicted. A clinical diagnosis of BPT was made and the patient started on steroids with some initial benefit.

Two months later he developed a further increase in breathlessness, despite continuing steroids. CXR showed increased intrapulmonary shadowing and PFT revealed a further decrease in DLCO to 52% of that predicted. Despite increased steroids the dyspnoea progressed, and serial CXR documented progressive fibrosis and development of cavitating lesions. Progressive hypoxia developed requiring increasing use of supplemental oxygen. Deterioration continued and the patient died of respiratory failure 10 weeks after chemotherapy had finished. Autopsy showed diffuse alveolar damage and pulmonary fibrosis consistent with BPT; there was no evidence of tumour.

© Cancer Research Campaign 1998

### Case II

A 44-year-old man presented with 6 weeks of leg weakness, urinary hesitancy and constipation. A clinical diagnosis of cauda equina syndrome was made on examination. CT scan showed destruction of the sacrum by a large retroperitoneal mass 12.8 cm in diameter, as well as two liver metastases each measuring 5 cm. The serum AFP was raised at 5163 IU $l^{-1}$, and biopsy of the retroperitoneal mass showed GCT. He was treated initially with radiotherapy to the sacrum (30 Gy in ten fractions). To avoid problems with early myelosuppression, and because of the extent of disease, he was treated with the BOP/VIP regimen (bleomycin, vincristine, cisplatinum/etoposide, ifosfamide, cisplatinum; Lewis et al, 1991). With the second cycle of BOP chemotherapy, the AFP level decreased to 43 IU $l^{-1}$. Two weeks after this cycle of chemotherapy (cumulative bleomycin dose 120 U), the patient became increasingly dyspnoeic. CXR showed patchy consolidation and he was started on empiric antibiotics for presumed infection. Over the following days, the dyspnoea increased and arterial oxygen saturations decreased. Examination revealed the development of a left pleural rub and right-sided crepitations. Bronchoscopy was negative for infection and CXR showed increasing patchy consolidation. Because of hypoxia he was transferred to the intensive care unit for intubation and ventilation. The hypoxia was progressive and unresponsive to treatment. Respiratory arrest and death occurred 3 months after initial diagnosis of GCT. Autopsy showed a severe degree of pulmonary fibrosis and extensive GCT with viable tumour involving the vertebral column, liver and retroperitoneum.

### Case III

A 43-year-old man presented with a short history of weight loss, anorexia, nausea and loin pain. A right testicular mass was palpable and confirmed by ultrasound scan. CXR revealed multiple pulmonary metastases. CT scans documented liver metastases, para-aortic lymphadenopathy and a solitary left frontal cerebral lobe metastasis. Serum HCG was markedly elevated at above 2 million IU $l^{-1}$. He proceeded to excision of the cerebral metastasis because of raised intracranial pressure and midline shift. A 4-cm haemorrhagic tumour was removed, and histology showed choriocarcinoma. Right orchidectomy was also performed and histology showed seminoma. Chemotherapy with cisplatin and etoposide (but not bleomycin) was started the day after craniotomy. At this time the patient developed dyspnoea thought to be secondary to the pulmonary metastases, and respiratory function progressively deteriorated over the following days requiring transfer to the intensive care unit for intubation and ventilation to maintain arterial oxygen saturations. CXR was unchanged from the time of presentation. Respiratory failure was prolonged and tracheostomy was performed. The patient slowly improved and, 3 weeks after onset, oxygen saturations of 95–98% were able to be maintained with inspired oxygen. The patient was then able to be weaned from the supplemental oxygen with arterial saturations remaining above 90%. During this period, chemotherapy was continued with cisplatin, methotrexate and vincristine. The HCG level decreased from 2 350 000 IU $l^{-1}$ at the start of chemotherapy to 18 344 IU $l^{-1}$ over this time. Serial CXR showed a slight decrease in the size of the metastases, but no other changes. He

**Table 3** Summary of trials assessing role of bleomycin in germ cell tumours

| Study | Patients | Regimen | Patient numbers | Chemotherapy | No evidence of disease after chemotherapy ± surgery | Disease-free survival | Relapse rate | Overall survival | Bleomycin toxicity |
|---|---|---|---|---|---|---|---|---|---|
| Loehrer et al | GCT. | PVP16 | 85 | Cisplatin 20 mg $m^{-2}$ days 1–5 | 88% | 69% | 23% | 86% | |
| (1995) | min/mod | vs | | Etoposide 100 mg $m^{-2}$ days 1–5 | | | | | |
| | disease | PVP16B | 86 | ±bleomycin 30 U $week^{-1}$ | 94% | 86% | 10% | 95% | Nil reported |
| | | | | 3 weekly ×3 | $P = 0.2$ | $P = 0.01$ | | $P = 0.01$ | |
| Levi et al | GCT. | PV | 108 | Cisplatin 100 mg $m^{-2}$ day 1 | 89% | 71% | 7% | | |
| (1993) | good | vs | | Vinblastine 6 mg $m^{-2}$ days 1+2 | | | | NS | |
| | prognosis | PVB | 112 | ±bleomycin 30 U weekly | 94% | 84% | 5% | | Two deaths |
| | | | | (CR+ two cycles, median 4) | $P= 0.29$ | | | | |
| De Wit et al | NSGCT. | EP | 195 | Cisplatin 20 mg $m^{-2}$ days 1–5 | 87% | 90% | 4% | | |
| (1997) | good | vs | | Etoposide 120 mg $m^{-2}$ days 1.3.5 | | | | NS | |
| | prognosis | BEP | 200 | ±bleomycin 30 U weekly | 95% | 94% | 4% | | Two deaths |
| | | | | 3 weekly × 4 | $P = 0.0075$ | | | | |
| Bosl et al | GCT. | EP | 82 | Cisplatin 20 mg $m^{-2}$ days 1–5 | 93% | | 12% | | |
| (1988) | good | | | Etoposide 100 mg $m^{-2}$ days 1–5 | | | | | |
| | prognosis | | | 3 weekly × 4 | | | | NS | |
| | | vs | | | | | | | |
| | | VAB-6 | 82 | Vinblastine 4 mg $m^{-2}$ day 1 | 96% | | 11% | | No deaths. 13 |
| | | | | Cyclophos 600 mg $m^{-2}$ day 1 | $P = NS$ | | | | patients |
| | | | | Dactinomycin 1 mg $m^{-2}$ day 1 | | | | | stopped bleomycin |
| | | | | Bleomycin 30 U day 1 | | | | | because of |
| | | | | Bleomycin 20 U $m^{-2}$ days 1–3 | | | | | DLCO |
| | | | | Cisplatin 120 mg $m^{-2}$ day 4 | | | | | |
| | | | | 4 weekly × 3 | | | | | |

NS, not sigificant was then started on BEP chemotherapy as he was no longer requiring supplemental oxygen and received three cycles of treatment, followed by two cycles of EP, over the following 4 months. A total dose of 270 U of bleomycin was given. The serum HCG continued to decrease until it reached a plateau of 40 IU $l^{-1}$, during the second cycle of EP chemotherapy. Coincident with this cycle of chemotherapy, he developed breathlessness and a cough with mild haemoptysis. Basal crepitations were present on examination and CXR showed decreased lung volume and bilateral fibrosis consistent with bleomycin toxicity. The extensive pulmonary metastases, although still present, had decreased in size. He was started on dexamethasone 16 mg $day^{-1}$ and his dyspnoea improved. The steroids were then weaned over a 2-week period. Further chemotherapy with cisplatin, methotrexate and vincristine was given as the HCG level had not normalized. One month later his condition deteriorated and steroids were reintroduced. Arterial oxygen saturations continued to decrease and supplemental oxygen was required. There was further deterioration and the patient died of respiratory failure 2 months after the clinical diagnosis of BPT was made. Consent for an autopsy was not obtained.

### Case IV

A 57-year-old man was investigated because of an 8-month history of backache and weight loss. A large retroperitoneal mass was found on CT scan and investigational laparotomy was performed. This showed haemorrhagic tumour extending from the epigastrium to the aortic bifurcation. The mass was thought inoperable and a biopsy was taken. Histology showed carcinoma with areas suggestive of seminoma. Both HCG (30 IU $l^{-1}$) and lactate dehydrogenase [LDH: 2000 IU $l^{-1}$ (< 180)] were raised. AFP was normal. Further investigation revealed an abnormality of the left testis on ultrasound scan. He was treated with four cycles of BEP chemotherapy, receiving a total of 330 U of bleomycin (bleomycin on day 15 cycle 1 was withheld because of thrombocytopenia). Tumour markers normalized after the first cycle of treatment. CT scan documented a decrease in the size of the retroperitoneal mass, with a maximum diameter of 8 cm at the completion of chemotherapy. Repeat testicular ultrasound showed patches of calcification in the previously abnormal area. Because of the large size of the residual mass and lack of definitive histology, he proceeded to resection of the mass and orchidectomy (2 months after the last bleomycin dose). This was a prolonged procedure of 9 h requiring both laparotomy and thoraco-abdominal incision. There was an estimated 9.5 l blood loss during the procedure, and two chest drains were left in situ at the conclusion of the operation. He received inspired oxygen concentrations of 34–40% during surgery and maintained arterial oxygen saturations above 90%. He was transferred to the intensive care unit with respiratory and renal compromise. CXR showed extensive alveolar shadowing of both lungs. Pulmonary artery catheterization showed high pressures consistent with fluid overload. He was treated with dopamine and frusemide infusion, renal dialysis and high-dose corticosteroids. Forty per cent inspired oxygen was required to maintain arterial oxygen saturation at 85–90%. Some days after admission to the intensive care unit, treatment was complicated by gastrointestinal haemorrhage, and two duodenal ulcers were found on endoscopy. The haemorrhage stabilized with fluid and blood support. Pulmonary function continued to deteriorate throughout this time, and the patient died from respiratory failure 3 weeks after surgery. Autopsy showed diffuse interstitial pulmonary fibrosis and hyperplasia of type II pneumocytes consistent with BPT. Three large duodenal ulcers were also identified. Histology of the surgical resection showed necrotic tissue only, with no evidence of residual tumour.

### Case V

A 67-year-old was treated with radiotherapy to the para-aortic lymph nodes (30 Gy in 15 fractions), for a stage IIB seminoma documented by orchidectomy and CT scan. One year later he presented with chest discomfort and cough. CXR showed a right hydropneumothorax, and a chest drain was inserted. LDH was raised at 618, although AFP and HCG were within normal limits. CT scan revealed a large posterior chest mass, with multiple lung nodules and mediastinal lymphadenopathy. Pleural biopsies showed recurrent seminoma. He was treated with BEP chemotherapy, receiving a total dose of 210 U of bleomycin (bleomycin was withheld cycle 1 days 8 and 15 because of hospital admission with neutropenic fever). Chemotherapy was complicated by grade III mucositis and prolonged neutropenia during the first cycle, for which he was treated with granulocyte colony-stimulating factor (GCSF) and empiric antibiotics. Repeat CT scan of the chest at the completion of chemotherapy showed residual thickening in the previous area of abnormality. Two months after completion of chemotherapy he returned with breathlessness and a dry cough. Examination revealed crepitations at the left lung base, and CXR showed patchy shadowing in this area. He was treated empirically with antibiotics, although afebrile, but his condition worsened. He became markedly hypoxic and was started on supplemental oxygen and corticosteroids. There was a transient improvement before continued deterioration. Repeat CXR showed extensive bilateral pulmonary shadowing consistent with BPT. Bronchoscopy, although planned, was not carried out because of worsening clinical state. The patient had a respiratory arrest and died. Permission for autopsy was denied. The diagnosis of BPT was made on clinical history, examination and radiological findings.

### DISCUSSION

The first reports of BPT described an incidence of 5% among patients receiving total cumulative doses of bleomycin below 450 U (Blum et al, 1973). For cumulative doses greater than 550 U the incidence of BPT increased to 17%, with an intermediate incidence of 13% for doses between 450 and 550 U. Fatal BPT occurred in less than 1% of patients at the lower dose but increased to above 10% for those who received over 550 U of bleomycin. The correlation of dose with toxicity was confirmed by Haas et al (1976), who found that for patients experiencing BPT the mean cumulative dose of bleomycin administered was significantly greater than that received by patients not developing toxicity. Fatal BPT can occur below the 450 U threshold, and the onset of BPT has been reported at cumulative doses lower than 100 U (Wilson et al, 1982; McLeod et al, 1987). Few patients receive cumulative doses above 360 U, and our present practice is to implement a ceiling of 270 U. The majority of our patients had been entered into current EORTC and/or MRC randomized trials over the study period. Within these trials, and for those patients not treated as part of a study protocol, bleomycin was given at a dose of 30 U either

 © Cancer Research Campaign 1998

as a 1-h intravenous infusion or as an intramuscular injection (for patients receiving outpatient treatment). In no case was bleomycin given as an intravenous bolus injection. The majority of protocols stipulated weekly doses of bleomycin (a total of 90 U per 3-week cycle), with a total of four cycles of treatment (i.e. a maximum of 360 U of bleomycin). The mean cumulative dose of bleomycin, in our patients, was lower than we expected from our current practice, but 43 of the patients reviewed had been treated on studies in the early 1990s that prescribed a single dose of 30 U of bleomycin with each cycle of chemotherapy, with a protocol ceiling dose of 120 U.

BPT has been reported in patients with renal impairment at low cumulative bleomycin doses (Bennett et al, 1980; Dalgleish et al, 1984; McLeod et al, 1987; Blayney et al, 1993). The terminal half-life of bleomycin is 2–4 h, and 70% of the administered dose is excreted in the urine within the first 24 h. The half-life can be greatly increased in the presence of renal impairment, with an exponential rise in the half-life as the creatinine clearance decreases below 35 ml $min^{-1}$. A bleomycin half-life of 21 h has been reported in a patient with a creatinine clearance of only 10.7 ml $min^{-1}$ (Crooke et al, 1977). Dose reduction is suggested for patients with creatinine clearances below 35 ml $min^{-1}$; however, the development of BPT has been noted in cases with lesser degrees of renal impairment in the absence of other risk factors (Dalgleish et al, 1984). The GFR of our fatal group was significantly lower than that of the non-fatal group, although there were comparable levels of renal impairment within the non-fatal group. No patient had a dose reduction on the basis of their GFR, as the values were within acceptable treatment limits.

Goldiner et al (1978) reported five fatal cases of BPT in patients post surgery. The onset of BPT after surgery was rapid, although the most recent administration of bleomycin in these patients had been 6–12 months previously. Goldiner et al postulated that the onset of BPT in these patients was related to the use of high inspired oxygen concentrations of 34–40% during surgery, and extensive fluid replacement (blood loss was often 10–15 units) during the 6- to 8-h operation. When the inspired oxygen concentration was kept between 22% and 24%, and fluid status was closely monitored with Swan–Ganz catheterization during operation, there were no further cases of BPT in patients undergoing similar procedures. The potentiation of BPT by high inspired oxygen concentration has been supported by animal studies (Tryka et al, 1984). In this review we have used surgery during or after chemotherapy as a surrogate measure of exposure to supplemental inspired oxygen before the development of BPT, as it was only during operations that the patients were exposed to supplemental oxygen according to clinical circumstances, although attempts were generally made to keep it low. There was no difference between the two groups in the frequency of operations performed, and therefore there was no discernible effect of supplemental oxygen on the frequency of fatal BPT.

The fifth fatal case received treatment with GCSF because of grade III prolonged neutropenia complicated by fever, during one cycle of chemotherapy. The possibility of synergy between bleomycin and GCSF in the aetiology of BPT has been raised (Matthews, 1993; Dirix et al, 1994) but has been refuted. In a large MRC/EORTC trial in which 263 patients receiving BEP or BOP/VIP chemotherapy for testicular teratoma were randomized either to receive GCSF or not, there was no evidence of an increase in BPT in the GCSF arm (Fossa et al, 1995).

All five fatal cases reviewed were over the age of 40, although only 47 of the 180 patients treated with bleomycin were in this age group. Blum et al (1973) reported a BPT incidence of 2–6% for each decade of life, which increased dramatically to 15% for those patients over 70 years (interestingly six of the seven deaths reported in his paper occurred in patients over age 40). The risk of developing fatal pulmonary toxicity is related to age, but we are concerned that the threshold of accelerating risk is below that traditionally quoted of 70 years (Blum et al, 1973; Haas et al, 1976), and that this threshold may be apparent in the fifth decade of life. The role of bleomycin, as part of curative treatment, in this subgroup of patients needs to be considered.

Our current standard therapy for good-prognosis GCT is four cycles of BEP chemotherapy. Einhorn et al (1989) reported that for the treatment of favourable prognosis GCT, three cycles of BEP chemotherapy were as effective as four. Decreasing the total administered dose of bleomycin (as well as that of cisplatin and etoposide) by omitting the fourth cycle did not compromise patient outcome, and was also associated with less treatment toxicity. This observation is currently being tested in a large prospective randomized MRC/EORTC trial (three vs four courses of BEP chemotherapy), although in this trial bleomycin is omitted from the fourth course of BEP (effectively a course of cisplatin and etoposide chemotherapy) in order to ensure an equal total dose of bleomycin (270 U) in both treatment arms.

A review of the literature indicates that four randomized trials have been performed assessing whether bleomycin could be withheld from standard regimens to lessen toxicity without compromising clinical response. The weight of evidence from these studies (Table 3) suggests that disease-free survival (DFS) of patients with good-prognosis disease decreases if bleomycin is omitted from these chemotherapy regimens. This is apparent with the individual trials showing either decreased initial response or an increased relapse rate if bleomycin is not used. The ECOG trial (Loehrer et al, 1995), which employed only three cycles of chemotherapy, showed the largest increase in relapse rate as a result of omitting bleomycin, and the trial was closed as a result of an interim analysis. In many centres this has led to the adoption of a policy of treating with three cycles of BEP as routine, or with four cycles of EP if bleomycin is contra-indicated. The EORTC trial (De Wit et al, 1997), the largest individual trial, shows a statistically significant decrease from 95% to 87% in attaining a disease-free state is bleomycin is withheld from primary chemotherapy, but only a 4% difference in DFS, and no significant difference in the overall survival of treated patients. This trend is supported by the other studies, although does not reach statistical significance, perhaps because of their smaller size. Most of the trials report similar relapse rates between their two treatment arms. The trial from the Memorial Sloan-Kettering Cancer Centre (Bosl et al, 1988) shows no significant difference in outcome between the two treatment arms, but the combinations being compared differed in other respects as well as the omission of bleomycin, hence the results are not directly comparable with the other trials.

Three of these four trials included both patients with seminoma or NSGCT in their analyses, although the numbers treated for seminoma were small. Previous studies have found cisplatin and etoposide chemotherapy to be highly effective treatment for advanced seminoma with a DFS of about 90% (Mencel et al, 1994), but the benefit of adding bleomycin to cisplatin and etoposide for this subgroup of GCT has not been studied. The peak age

© Cancer Research Campaign 1998

incidence of seminoma is substantially different from that of NSGCT. 35–39 years vs 25–29 years respectively (Forman and Moller, 1994), and therefore is more prevalent among the older GCT patients, i.e. the subgroup of patients that we feel are at greater risk of developing fatal BPT.

In our view, for that group of patients with GCT over 40 years of age, particularly those with a GFR at the lower end of the normal range, it is preferable to accept the possibility of a reduction in DFS by perhaps 4% in order to avoid a risk of fatal BPT that may exceed 10%. We have therefore discontinued the routine use of bleomycin in these patients, with good prognosis disease, for whom our standard regimen comprises four cycles of treatment with cisplatin and etoposide.

### REFERENCES

Bennett WM, Pastore L and Houghton DC (1980) Fatal pulmonary bleomycin toxicity in cisplatin-induced acute renal failure. *Cancer Treat Rep* **64**: 921–924

Blayney DW, Goldberg DA, Leong LA, Margolin KA, Burke JS and Doroshow JH (1993) High risk germ cell tumours in men: high response rate and severe toxicity with cisplatin, vinblastine, bleomycin, and etoposide. *Cancer* **71**: 2351–2357

Blum RH, Carter SK and Agre K (1973) A clinical review of bleomycin – a new antineoplastic agent. *Cancer* **31**: 903–914

Bosl GJ, Geller NL, Bajorin D, Leitner SP, Yagoda A, Golbey RB, Scher H, Vogelzang NJ, Auman J, Carey R, Fair WR, Herr H, Morse M, Sogani P and Whitmore W (1988) A randomised trial of etoposide + cisplatin versus vinblastine + bleomycin + cisplatin + cyclophosphamide + dactinomycin in patients with good-prognosis germ cell tumours. *J Clin Oncol* **6**: 1231–1238

Comis RL (1992) Bleomycin pulmonary toxicity: current status and future directions. *Semin Oncol* **19** (suppl. 5): 64–70

Crooke ST, Comis RL, Einhorn LH, Strong JE, Broughton A and Prestayko AW (1977) Effects of variations in renal function on the clinical pharmacology of bleomycin administered as an IV bolus. *Cancer Treat Rep* **61**: 1631–1636

Cytel (1989) *StatXact User Manual*. Cytel Software Corporation: Cambridge, MA, USA

Dalgleish AG, Woods RL and Levi JA (1984) Bleomycin pulmonary toxicity: its relationship to renal dysfunction. *Med Pediatr Oncol* **12**: 313–317

De Wit R, Stoter G, Kaye SB, Sleijfer DT, Jones WG, ten Bokkel Huinink WW, Rea LA, Collette L and Sylvester R (1997) The importance of bleomycin in combination chemotherapy for good-prognosis testicular non-seminoma: a randomised study of the EORTC genitourinary tract cancer co-operative group. *J Clin Oncol* **15**: 1837–1843

Dirix LY, Schrijvers D, Druwe P, Van Den Brande, Verhoeven D and Van Oosterom AT (1994) Pulmonary toxicity and bleomycin (letter). *Lancet* **344**: 56

Einhorn LH, Williams SD, Loehrer PJ, Birch R, Drasga R, Omura G and Greco FA (1989) Evaluation of optimal duration of chemotherapy in favorable-prognosis disseminated germ cell tumours: a southeastern cancer study group protocol. *J Clin Oncol* **7**: 387–391

Forman D and Moller H (1994) Testicular cancer. *Cancer Surveys* **19/20**: 323–341

Fossa S, Kaye SB, Mead GM, Cullen M, De Wit R, Bodrogi I, van Groeningen C, Sylvester R, Stenning S, Vermeylen K, Lallemand E and de Mulder P (1995) An MRC/EORTC randomised trial in poor prognosis metastatic teratoma, comparing treatment with/without filgrastim (G-CSF). *Proc Am Soc Clin Oncol* **14**: 656

Goldiner PL, Carlon GC, Cvitkovic E, Schweizer O and Howland WS (1978) Factors influencing postoperative morbidity and mortality in patients treated with bleomycin. *Br Med J* **1**: 1664–1667

Haas CD, Coltman CA, Gottlieb JA, Haut A, Luce JK, Talley RW, Samal B, Wilson HE and Hoogstraten B (1976) Phase II evaluation of bleomycin: a southwest oncology group study. *Cancer* **38**: 8–12

Levi JA, Raghaven D, Harvey V, Thompson D, Sandeman T, Gill G, Stuart-Harris R, Snyder R, Byrne M, Kerestes Z and Margrie S (1993) The importance of bleomycin in combination chemotherapy for good-prognosis germ cell carcinoma. *J Clin Oncol* **11**: 1300–1305

Lewis CR, Fossa SD, Mead G, ten Bokkel Huinink B, Harding MJ, Mill L, Paul J, Jones WG, Rodenburg CJ, Cantwell B, Keizer HJ, van Oosterom A, Soukop M, Splinter T and Kaye SB (1991) BOP/VIP – a new platinum-intensive chemotherapy regimen for poor prognosis germ cell tumours. *Ann Oncol* **2**: 203–211

Loehrer PJ, Johnson D, Elson P, Einhorn LH and Trump D (1995) Importance of bleomycin in favorable-prognosis disseminated germ cell tumours: an Eastern Co-operative Oncology Group trial. *J Clin Oncol* **13**: 470–476

McLeod BF, Lawrence HJ, Smith DW, Vogt PJ and Gandara DR (1987) Fatal bleomycin toxicity from a low cumulative dose in a patient with renal insufficiency. *Cancer* **60**: 2617–2620

Matthews JH (1993) Pulmonary toxicity of ABVD chemotherapy and G-CSF in Hodgkin's disease: possible synergy (letter). *Lancet* **342**: 988

Mencel PJ, Motzer RJ, Mazumdar M, Vlamis V, Bajorin DF and Bosl GJ (1994) Advanced seminoma: treatment results, survival, and prognostic factors in 142 patients. *J Clin Oncol* **12**: 120–126

Sleijfer DT and Mulder NH (1997) Treatment of advanced seminoma: an update. *Anti-Cancer Drugs* **8**: 107–112

Tryka AF, Godleski JJ and Brain JD (1984) Differences in effects of immediate and delayed hyperoxia exposure on bleomycin-induced pulmonary injury. *Cancer Treat Rep* **68**: 759–764

White DA and Stover DE (1984) Severe bleomycin-induced pneumonitis: clinical features and response to corticosteroids. *Chest* **86**: 723–728

Wilson KS, Worth A, Richards AG and Ford HS (1982) Low-dose bleomycin lung. *Med Pediatr Oncol* **10**: 283–288

© Cancer Research Campaign 1998